# Current progress in hepatic tissue regeneration by tissue engineering

**DOI:** 10.1186/s12967-019-02137-6

**Published:** 2019-11-21

**Authors:** Vahid Hosseini, Nazila Fathi Maroufi, Sepideh Saghati, Nahideh Asadi, Masoud Darabi, Saeed Nazari Soltan Ahmad, Hosseini Hosseinkhani, Reza Rahbarghazi

**Affiliations:** 1 Stem Cell Research Center, Tabriz University of Medical Sciences, Imam Reza St., Golgasht St., Tabriz, 5166614756 Iran; 2 Department of Biochemistry and Clinical Laboratories, Faculty of Medicine, Tabriz University of Medical Sciences, Tabriz, Iran; 3 Department of Tissue Engineering, Faculty of Advanced Medical Sciences, Tabriz University of Medical Sciences, Tabriz, Iran; 4 Department of Nanotechnology, Faculty of Advanced Medical Sciences, Tabriz University of Medical Sciences, Tabriz, Iran; 5 Student Research Committee, Tabriz University of Medical Sciences, Tabriz, Iran; 6 Innovation Center for Advanced Technology Matrix, Inc., New York, NY 10029 USA; 7 Department of Applied Cell Sciences, Faculty of Advanced Medical Sciences, Tabriz University of Medical Sciences, Tabriz, Iran

**Keywords:** Hepatic regeneration, Tissue engineering modalities, Stem cells

## Abstract

Liver, as a vital organ, is responsible for a wide range of biological functions to maintain homeostasis and any type of damages to hepatic tissue contributes to disease progression and death. Viral infection, trauma, carcinoma, alcohol misuse and inborn errors of metabolism are common causes of liver diseases are a severe known reason for leading to end-stage liver disease or liver failure. In either way, liver transplantation is the only treatment option which is, however, hampered by the increasing scarcity of organ donor. Over the past years, considerable efforts have been directed toward liver regeneration aiming at developing new approaches and methodologies to enhance the transplantation process. These approaches include producing decellularized scaffolds from the liver organ, 3D bio-printing system, and nano-based 3D scaffolds to simulate the native liver microenvironment. The application of small molecules and micro-RNAs and genetic manipulation in favor of hepatic differentiation of distinct stem cells could also be exploited. All of these strategies will help to facilitate the application of stem cells in human medicine. This article reviews the most recent strategies to generate a high amount of mature hepatocyte-like cells and updates current knowledge on liver regenerative medicine.

## Background

**Fig. 1:**
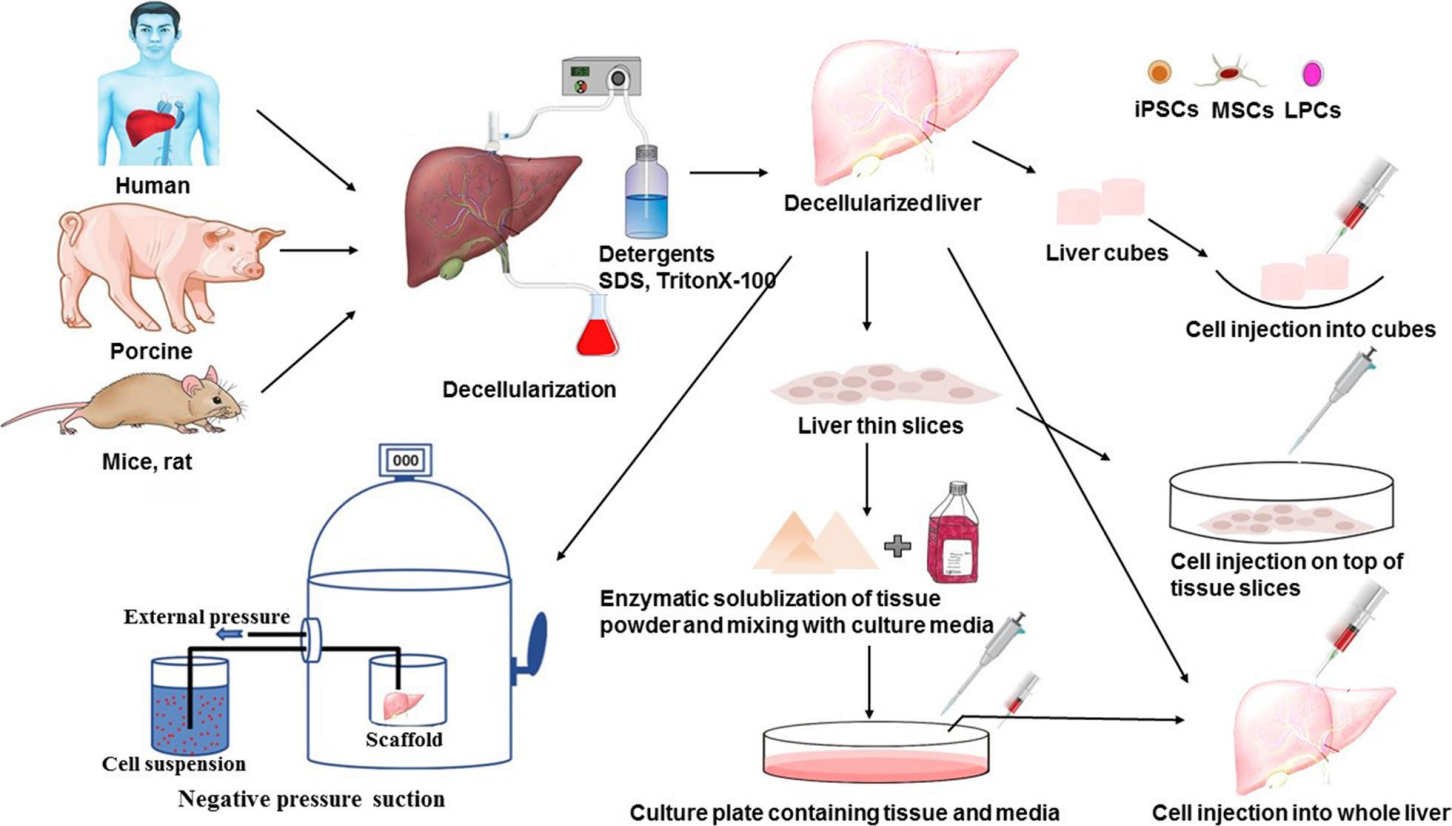
Hepatic tissues from various sources such as human, porcine and rat underwent decellularization using detergents. Repopulation of the decellularized liver scaffold is performed through various routes. Decellularized liver scaffold is chopped to cubes or thin slices then recellularized by microinjection. Whole-organ reseeded by exerting negative pressure suction. Various cell types such as induced pluripotent stem cells (iPSCs), mesenchymal stem cells (MSCs) or liver progenitor cells (LPCs) are used for recellularization

**Fig. 2:**
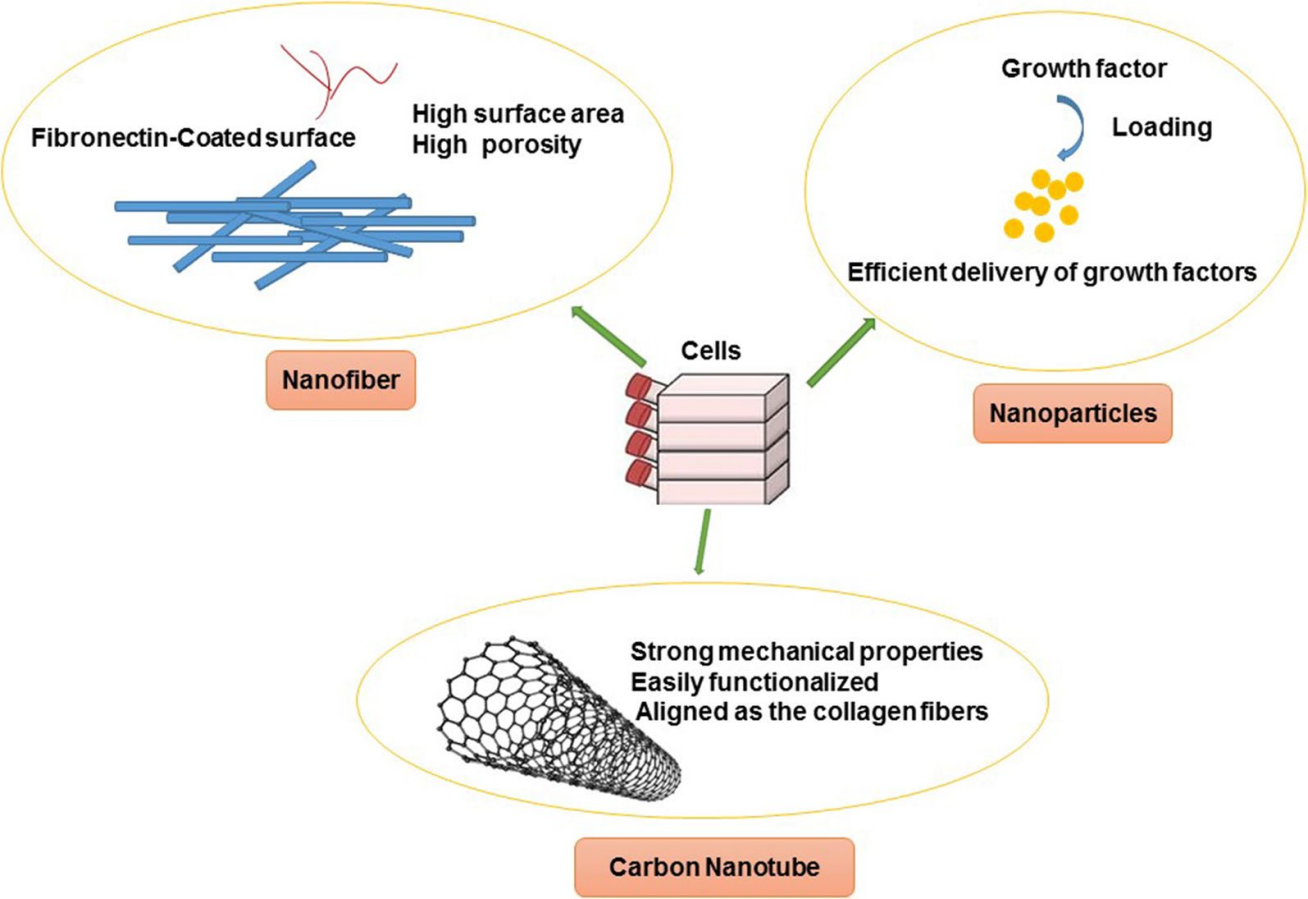
The impact of various nanostructures on hepatic differentiation. Nanofibers (high surface area, high porosity), nanoparticles (efficiently growth factors delivery) and carbon nanotube (mechanical properties, easily functionalized, aligned as collagen

The liver is the largest gland of the body, which normally weighs about 1.5 kg in adults and divided into a large right lobe and a smaller left lobe [1]. Each lobe is further divided into lobules, which are the functioning units of the liver. The lobule is consisting of a hexagonal row of hepatocytes. Primary hepatocytes constitute 60–80% of the liver mass and play many important functions in our body. The main functions played by the liver include (a) bile production and secretion; (b) excretion of bilirubin, cholesterol, hormones, and drugs; (c) metabolism of fats, proteins, and carbohydrates; (d) enzyme activation; (e) storage of glycogen, vitamins, and minerals; (f) macromolecules and protein synthesis (i.e. Alb and bile acids); and (g) detoxification [2]. Detoxification is a critical liver-specific function [3]. Exogenous and endogenous substances are detoxified in the liver by two main mechanisms, phase I and phase II biotransformation [4, 5]. Hepatocyte-based hepatotoxicity testing is useful in the rapid screening of chemicals and in the mechanistic evaluation of toxicological phenomena. A large amount of natural and synthetic chemicals are hepatotoxins. In many cases, the hepatotoxicity is due to an impaired hepatocyte metabolism and conversion of inert and non-toxic compounds into highly reactive metabolites. The hepatocytes are usually the first cell types that are damaged upon hepatotoxic insult [6]. The loss of liver functions such as detoxification, metabolism, and regulation causes life-threatening complications, including kidney failure, encephalopathy, cerebral edema, severe hypotension and susceptibility to infections culminating in multiple organ failure. Hepatocytes-based screening can be used to characterize the metabolic fate of compounds and whether metabolism contributes to toxicity. Therefore, approaches to screen hepatocytes are of great biomedical importance. The bioartificial engineering liver constructs have been recently developed [7, 8]. The efforts in the modern liver tissue engineering field mainly include: (1) creating a whole, implantable, and functional tissue-engineered liver constructs; (2) establishing bioartificial liver systems to sustain liver patient’s lives before liver transplantation, establishing in vitro hepatocyte-based model; (3) establishing a culture model for drug metabolism/toxicity screening for drug discovery and (4) for basic researchers of liver regeneration, disease, pathophysiology and pharmacology (Figs. 1, 2).

**Fig. 3:**
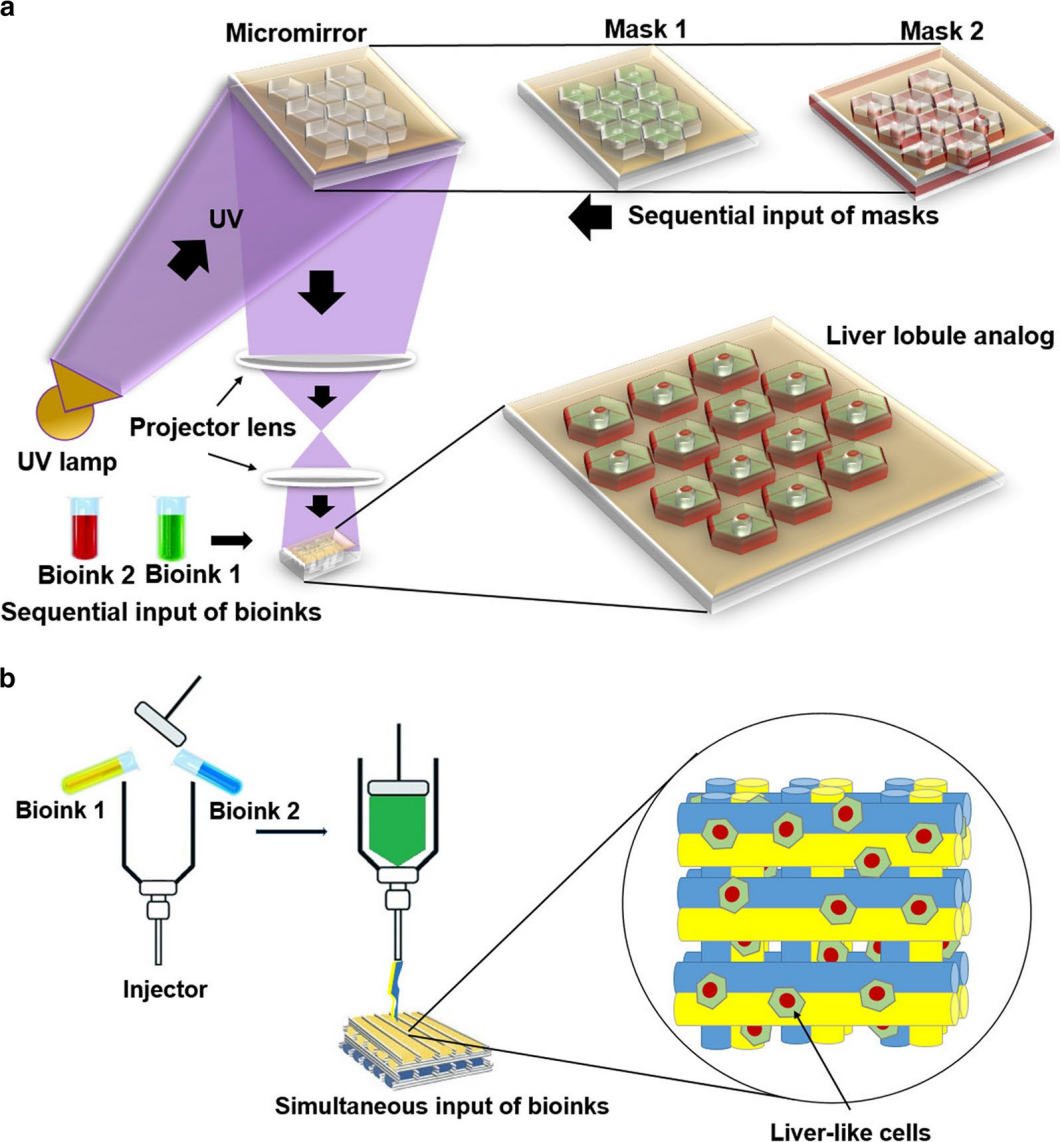
Schematic overviews of multi-material 3D bioprinting approach. Sequential 3D bioprinted hepatic lobule-like structures (**a**). Simultaneous deposited and dual fabricated 3D structures (**b**)

**Fig. 4:**
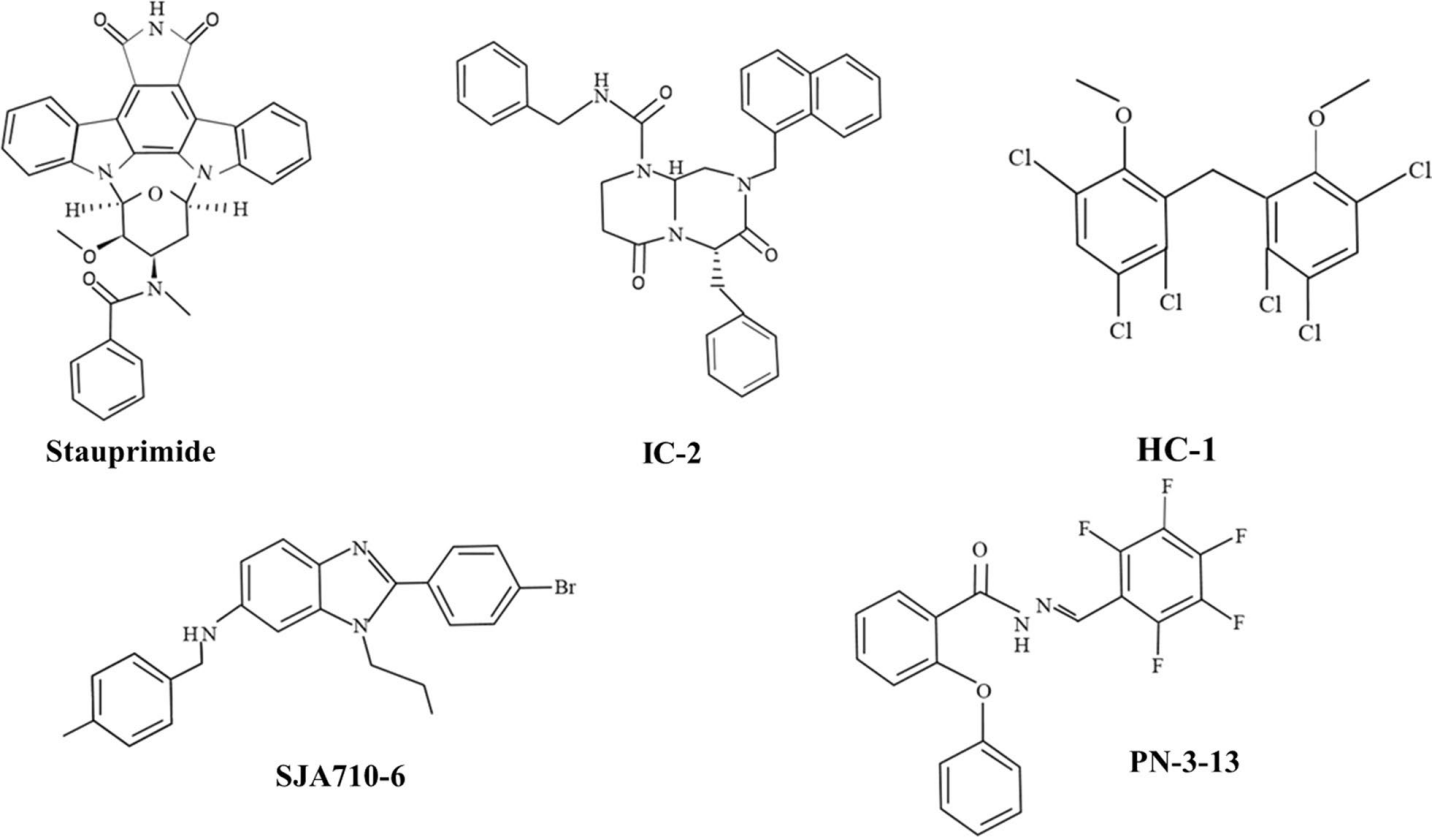
A summary of methods used to hepatic differentiation of stem cells using intracellular signaling pathway. *DMSO* dimethyl sulfoxide, *DKK-1* Dikkopf-related protein-1, *HNF-3β* hepatocyte nuclear factor 3-β, *PrP-1* poly-ADP-ribose polymerase-1

The liver is an important organ because of critical metabolic functions such as protein synthesis and xenobiotic biotransformation [9]. The liver possesses remarkable regenerative capacity but viral infection, toxic compounds, and cancer resulted in vast injury and liver dysfunction, if continued, leading to end-stage liver pathologies and hepatic failure which can threaten life. In the case of liver transplantation immunological incompatibility between the donor and recipient, however, limits the application of this procedure [10–12]. Transplantation at cell levels such as hepatocyte provides several benefits over liver transplantation, can be performed several times and offer a less invasive alternative to whole liver transplantation, but it demonstrates low levels of engraftments [13]. Therefore, researchers bring forward to test novel tissue engineering approaches aiming to fabricate 3D hepatic tissue in microscale size or whole bioengineered liver synthesis to restore damaged liver function even after massive injury and resolve the donor shortage problem. Over the past years, the liver bioengineering field has experienced significant progress in the area of cell engineering, biomaterials fabrication, and tissue architecture to recapitulate transplantable microscale liver tissue and whole organ bioengineering as well [14]. Genetic engineering strategies and hepatic differentiation of stem cells are also under tight investigations [15, 16]. A wide range of cells from hepatocyte primary cells to hepatocyte-like cells that generated from hiPSCs constitute the promising source of stem cells which can be expanded in a high quantity that has appropriate compatibility with the host immune system. Considerable efforts have been dedicated to evaluating decellularized liver scaffolds in the construction of natural 3D extracellular matrix to better integration of mechanical and chemical signals for stem cell differentiation and maturation (Table 1). In addition, as a new 3D culture system, the perfusion-based culture approaches have been emerged and showed better potential to model in vivo tissue microenvironment state. In spite of conventional 2D monolayer systems, the cells can pile on top of each other in perfusion based culture system which in turn leads to improve material exchange and cell to cell communication, especially in prolonged culture periods [17, 18]. Besides liver tissue engineering, various nanomaterials and nanoparticles are used in in vitro hepatic differentiation of stem cells. Nanomaterials can emulate native liver ECM and nanoparticles due to their biodegradability and good biocompatibility are used to direct delivery of hepatogenic small molecules, growth factors, cytokines and proteins to stem cells [19]. 3D bioprinting system, as a sophisticated engineering methodology, has been improved to solve the issues related to conventional 2D culture technique, by providing clues essential for the dynamic of distinct cell types in the context of in vivo milieu. Evidence point that cell performance is enhanced by promoting the juxtacrine cell-to-cell interaction in the massive scale and 3D printing system has the potential to fabricate the distinctive constructions applicable to target tissues and organs [20–22]. In the 3D printing system, a computer identifies a 3D spatial model of the target organ and cuts it into sequences of 2D slices, which is then assembled from bottom to the top. This system is able to print and copy any preferred shapes with the facility to direct cell distribution, scaffold pore size, interconnectivity, and geometry. It is noteworthy that if the operator error is reduced, it can contribute to the formation of optimal uniform shapes on a large scale structure [23–25]. Apart from the mentioned approaches, some valuable steps have been taken in the application of small molecules and micro-RNAs as well as genetic manipulation in hepatic differentiation of stem cells. Small molecules have been reported to play significant roles in developmental processes through modulation of some signaling pathways involved in cell fate determination such as Wnt, Notch, and FGF during cell differentiation [26]. The application of small molecules in in vitro hepatic induction protocols demonstrated promising results through the assessment of hepatic markers [16]. MicroRNAs are constitutively or transiently expressed during differentiation and involved in cell fate determination programs of stem cells and researchers recently investigated the impact of microRNAs alone or in combination with each other as well suppression of some of them on hepatic differentiation of stem cells [27, 28]. In parallel, it has been shown that transcription factors such as FGF, HGF, Wnt, BMP, RA, and TGFβ to correlate with effectors influencing liver development. Therefore, it has been expected that transferring and integration, overexpression or knockdown of one or more than one transcription factors in stem cells can be able to enhance expression hepatic commitment markers [29]. In this review, the authors attempt to discuss the most recently addressed approaches aiming to improve the hepatic differentiation rate by different tissue engineering modalities as a new avenue for transplantation after hepatic failure or as therapy for liver regeneration and replacement (Figs. 3, 4).

**Table 1:** Different methodologies used for recellularization of acellular hepatic tissue by using different cells

Model	Decellularization procedure	Evaluation of ECM	Recellularization procedure	Recellularized cell	Results	Day of cells survival on ECM	Refs
Human	PBS: overnight 0.01% SDS: 4 h 0.1%, 0.2% and 0.5% SDS: 1 h for each 1% tritonX-100: 15 min PBS: 5 min	99% of DNA removed, 60% of collagen and 40% of GAGs preserved	Decellularized slices digestion by 1 mg/ml pepsin in 10 mM HCl: 24 h, RT, centrifuge, supernatant coated on plates Hepatic differentiation	hiPSC	Alb production at day 4, higher expression of stage-specific markers compared to control cells cultured on Matrigel and collagen 1	20 days	[121]
Porcine	1% tritonX-100 perfusion: 3 h, 200 ml/min 1% SDS perfusion: 6 h, 200 ml/min 1% tritonX-100 perfusion: 3 h 20 l DW 40 l PBS Liver discs lyophilization	–	Rehydration of liver disk with IMDM: overnight, 370c Cell seeding on disk using negative pressure suction and hepatic induction: 14 day	Human umbilical cord mesenchymal stem cell	ND	More than 14 days	[217]
Porcine	Trypsin–EDTA, 1 h, 370c 1% tritonX-100, 0.1% ammonium hydroxide, 8 h DW, overnight 0.1% PAA, 4% ethanol, 2 h	98% dsDNA (< 50 ng DNA fragment < 200 bp), all cell nuclei removed, GAGs proteins, HGF, bFGF and EGF preserved, low chemoattraction	Tissue powder solubilization by Acetic acid and gelation by pepsin, coating on plate	HepG2, liver primary cells	The high amount of urea, alb production compared to control cells cultured on Collagen 1	More than 7 days	[120]
Human HepaRG cell	DE induced cells were differentiated to the hepatic lineage Cell culture plate washing with DW Incubation with DW: 45 min, 370c Wash with DW: 1 time	DAPI and filamentous actin staining confirmed cell removal	Cell lines cultured on acellular matrix in hepatic induction was done using growth factors	ESC-WA07, ESC-H9, hiPSC	Response to differentiation was varied among cells, more than 90% of WA07 and H9 demonstrated the liver function	Around 20 days	[123]
Rat	DW: 5 ml/min, 40 times of liver volume 1% TritonX-100 and 0.1% ammonium hydroxide: 50 times of liver volume DW	98.9% of DNA removed, 85% of GAGs, 52% of proteins and 71% of collagen was conserved, 60% of ECM component conserved after 28 days treatment with PBS solution	Acellular liver powder digestion by trypsin and HCl, the coating on 24 well plates, overnight gelation Cell seeding, hepatic induction by growth factor	Human BM-MSC	After 28 days the treated cell exhibit hepatocyte phenotype resulted cells could uptake LDL and were AFP, HNF4, CYP, and Alb positive	More than 28 days	[218]
Porcine	0.01% SDS perfusion: 150 l 0.1% SDS perfusion: 150 l 1% SDS perfusion: 50 l 1% tritonX100: 50 l DW: 100	Mesh structures were preserved, no remaining of the nucleus and intact cells, the scaffold was rich in GAGs	The ECM powder homogenized in PBS and treated with collagenase 1 to form a gel. 1 ml of the resulting gel mixed with 1 * 106 cells and hepatic induction carried out in the absence of growth factors	Bone marrow-derived-MSCs	Glycogenesis and Alb production	14 days	[124]
Ferret	DW: 2 l, 6 ml/min TritonX-100, 0.1% ammonium hydroxide: 4 l Dw: 8 l	–	Acellular tissue minced in small discs with 8 mm diameter and placed in 48 well plates. 3–5 * 105 cells suspended in seeding solution and then transferred on top of discs. Hepatic induction using growth factors	Human liver progenitor cells	Expression pattern similar to hepatocytes (Alb+, CK19− and EpCAM−) and duct (Alb−, CK19+, and EpCAM+). The cells were negative for Alb and positive for GNF4α indicating the maturation of hepatocyte-like cells, detoxification activity	3 weeks	[122]
Porcine	Heparinized PBS: 6 h SDS 0.1%: 72 h PBS: 12 h 0.1% PAA: 0.5 h	99.4% of DNA removed, 65% of collagen and GAGs, as well as more than 40% of growth factors, was preserved	Decellularized cubic liver powder solubilization in 3 mg pepsin/0.1 M HCl: 72 h, RT, 120 rpm, the resulting solution was concentrated tenfolds by addition of 1/10 of the total volume of 1 M NaCl and PBS10X. Preparation of 0%, 2%, 5%, 10% and 20% of ECM in hepatic differentiation media	Porcine IPSCs	Alb protein expression and secretion into the media increased fourfold and twofold, respectively, in comparison to control groups	20 days	[14]
Rat	Heparinized saline: 20 ml 0.1% SDS: 3 ml/min, 15 h PBS: 12 h 0.1% PAA: 15 min	The amount of DNA was below a detectable level, more than 60% of collagen and all of the GAGs maintained, methylene blue staining revealed no vascular tree destruction	The recellularization carried out 4 times through injection of 5 * 106 cell/0.15 ml/min with 30 min intervals. Hepatic induction without using growth factors	Hepatocyte induced porcine IPSCs	Alb and urea secretion	5 days	[14]
Rat	Saline: 20 ml DW: 5 ml/min, 40 times of liver volume 1% tritonX-100 and 0.1% NaOH: 50 times of liver volume DW: wash out the detergent	98.9% of DNA was declined, 47% of HGF, 48% of bFGF as well as fibronectin and laminin preserved	The tissue minced to 8 mm disks and placed on 24 well plates. Overnight incubation by Hepatocyte media, 10 * 6 cells in 20 µl media pipetted to scaffold and after 20 min 10 of scaffolds transferred to t25 flask placed on a shaker	Human iPSC-derived hepatocyte	The cell proliferation was increased, average gene expression of CYP2C9, CYP3A4 and HMGCR 5 times increased, fetal liver markers AFP and CYP3A7 decreased during the culture period	14 days	[118]
Porcine	1% SDS: 36 l, 200 ml/min, 3 h DW: 200 ml/min, 3 h 1% SDS: 3 l, 200 ml/min, 3 h DW: 200 ml/min, 3 h Repetition of previous steps 1% Triton-100: 36 l DW: 36 l, 200 ml/min PBS: 36 l, 200 ml/min	Cellular components and nuclei were removed, reticular collagen fibers were observed	The 100 µl of MSCs spheroids suspension pipetted on top of DLSs under negative pressure suction following overnight incubation in culture medium at 370c. hepatic induction was done by growth factors	Bone-marrow-derived MSCs	Efficient expression of Albp-ZsGreen, Alb, drug-metabolizing enzymes, and enzymes related to fat and amino acid metabolism as well as higher secretion of urea than 2D cultures	23 days	[219]
Rat	2% sodium deoxycholate: 2 ml/min, 4 h DW: detergent washing 3% triton-100: 4 h DW containing 0.02% sodium azide and 5 mM EDTA: 72 h 1% PAA: 1 h PBS: 500 ml	99.3% of DNA is removed, the amount of collagen and elastin were more than that assessed for wet tissue, 60 and 15% respectively. 17% of GAGs, 0.1% of cytokines were preserved, the intact cells and nuclei were removed	Pretreatment of whole liver scaffolds with collagen and GAGs for 60 min followed by overnight treatment by DMEM. DMEM was exchanged with liver fetal cell medium and the recellularization of livers with 44 and 73*106 cells was completed for 7 days and stopped at day 11	Human fetal liver progenitor cells: hFL4TERT and SV40	The recellularized cells were viable in four of six livers until the end of experiments. A low expression or lack of liver function markers such as Alb, CYP450, and CKs. The cells were positive for human mitochondria and endothelial markers	11 days	[131]

## Cell sources for liver regeneration

To generate donor-free and expandable hepatocyte cells source, several types of cells are exploited in liver tissue engineering. Based on previous studies in this area, these cells include a primary culture of hepatocytes, ESCs, iPSCs, and MSCs. ESCs are originated from the inner cell mass of blastocysts. To obtain iPSCs, adult somatic cells are genetically manipulated and reprogramed. For this propose, expression of pluripotency factors such as Oct4, Sox2, c-Myc, and klf4 is stimulated in the target cells [30]. It should be noted that MSCs are commonly isolated from almost all connective tissues mainly bone marrow medullary niche and adipose tissue. Using primary cell culture strategy, expanded hepatocytes retain and preserve specific functions such as drug metabolism activity and etc. which are comparable to the in vivo condition; however, prolonged in vitro expansion may lead to cell survival decrease and cell-specific function removal. In addition, it should not be forgotten that In addition, these cells should be freshly prepared from the patients to prevent immune cell reactivity and transplant rejection. To circumvent these pitfalls, great efforts have been devoted to improving functional behavior in the primary culture of hepatocytes. For instance, the application of 2D, 3D culture models, and perfusion-based microfluidic systems are at the center of attention [31, 32].

**Fig. 5:**
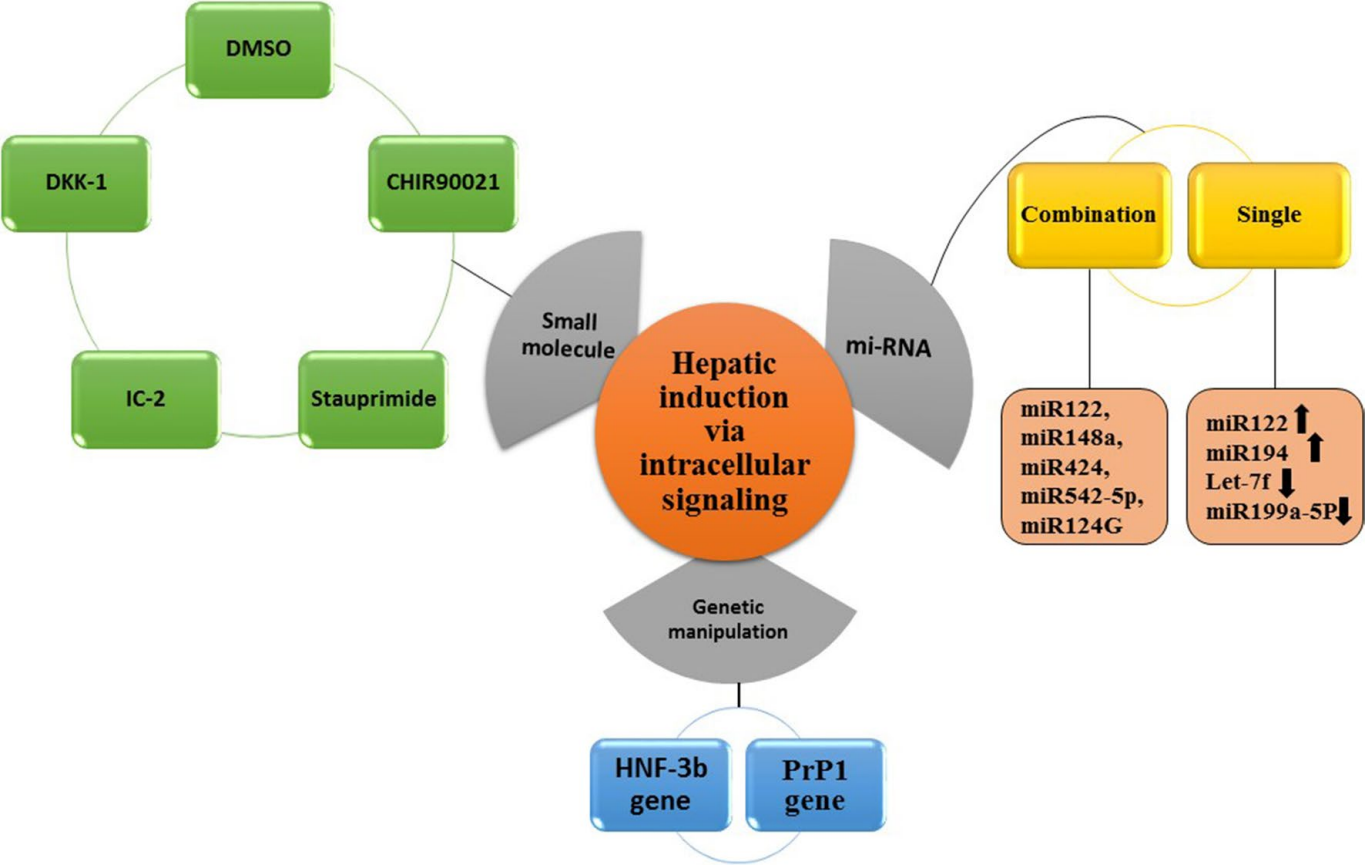
Some of the chemical structures related to small molecules are used commonly for hepatic-like phenotype induction from progenitor cells

Perfusion-based systems are able to simultaneously replace fresh medium with the exhausted medium and continuously eliminate metabolic byproducts from the culture condition. Several experiments have highlighted an enhanced of hepatic cells function expanded in 2D, 3D culture models and perfusion-based systems, indicated by the up-regulation of liver function factors. ESCs and iPSCs possess high self-renewal capability that facilitates trans-differentiation into multiple cell lineages under specific conditions. It has been shown that the presence of specific growth factors, cytokines, and small molecules could increase differentiation properties. For instance, in a recent study, it was shown that ESCs could differentiate into hepatocyte-like cells in a stepwise manner using small molecules LY294002, touted as definitive endoderm inducer, bromo-indirubin-3′-oxime, odium butyrate, dimethyl sulfoxide and growth factor activin A. Among these factors, bromo-indirubin-3′-oxime, odium butyrate could dictate cells to acquire hepatoblast-like phenotype while dimethyl sulfoxide could accelerate orientation of progenitor cells toward mature hepatocyte-like cells. Differentiated cells have the ability to express hepatic cells specific factors and products such as urea, Alb and cytochrome p450 enzymes. In addition drug detoxification activity was similar to the human primary hepatocytes [33]. Scientific reports have pointed that iPSCs have some advantages over the ESCs. The use of iPSCs does not provoke immune cell activity and there are ethical issues exist surrounding the transplantation of ESCs. Recently, Rashidi et al. differentiated human iPSCs cells, lines FSPS13B and P106, into definitive endoderm cells by using activin A and bFGF followed by cell maturation into hepatocytes in the presence of HGF and OSM in a spheroid culture system. These spheroids were functional for more than 1 year and showed hepatic cells function and expressed maturation markers. It was found that these spheroids can partially support liver function in hepatectomized animal model after subcutaneous or intraperitoneal transplantation [34]. Similar to iPSCs and ESCs, MSCs also have shown a high hepatic differentiation potential either in vivo or in vitro model [35]. Bone marrow-derived MSCs demonstrated an enhanced expression of hepatocyte-specific markers and exhibited hepatocellular function after introduction to the liver decellularized scaffold in the presence of EGF and HGF (Fig. 5).

In another study, iPSCs-derived MSCs and iPSCs were successfully transplanted into a hepatectomized rat to reduce hepatic injury. The cells were successful tracked in the rat hepatic tissue even after 2 months. The reduction of systemic bilirubin and increase of liver-specific markers such as Alb, cytokeratin-18, and α-fetoprotein were reported [36]. All these findings highlight the potency of ESCs, iPSCs, and MSCs to differentiate into hepatocyte-like cells. It seems that an inherent capacity of stem cells to circumvent problems related to post-transplantation hosts makes these cells superior to hepatocyte primary cells in the regeneration of hepatic tissue. Nevertheless, we must not forget that stem cells are not a magic bullet for the regeneration of hepatic tissue. Establishing precise criteria and measurement of differentiation capacity in the hepatic tissue must be carefully monitored after stem cell introduction.

### Multicellular interactions in the hepatic tissue

Although in vitro culture models demonstrate hepatic tissue function, but these models could not completely restore the vital organ function such as metabolism and synthesis. All these systems mainly concentrate on the hepatocyte cells expansion. To achieve fully functional liver tissue, we need all cell types to exist in the liver, including PCs and NPCs cells [37]. Liver tissue is composed of NPCs such as Kupffer cells, endothelial cells and hepatic stellate cells that perform liver functions in collaboration with hepatocytes. The space between the liver NPC and hepatocyte is filled with a protein-rich material so-called Space of Disse. This space plays an important role in liver function through the regulation of materials and nutrients exchanging between endothelial cells and hepatocytes [38]. The conventional culture models of hepatocytes expansion are unable to mimic normal hepatic tissue microarchitecture. The function of hepatocytes correlates with the promotion of hepatocyte–hepatocyte, hepatocyte–ECM and hepatocyte–NPCs interaction. The lack of reciprocal interactions, as seen in conventional culture systems, leads to hepatocyte rapidly lose hepatocyte-specific bioactivities [37]. Due to the important role of NPC in liver function, recently magnificent efforts are ongoing to develop culture systems that incorporate multiple cell types including PCs and NPCs in hepatocyte culture [39]. It has shown that the co-culture system consisted of hepatocytes and NPCs promoted hepatocytes function through the initiation of hepatocyte-different cell interaction both in juxtacrine and paracrine manners. Calling attention, each cell of liver tissue plays vital roles in maintaining liver function [40]. By using a culture system composed of three rat liver cells including hepatocytes, Kupffer and endothelial cells on a polymer surface, A Space of Disse-like structure was reconstructed. It was shown that all these cells proliferated and preserve their morphologies in a 3D system compared to the conventional 2D culture. The expression of Alb and Cytochrome 450 was higher when stiffness of the scaffold was similar to the normal liver [41]. In another study, the hepatic differentiation was shown in a co-culture system using human iPSCs and adipose microvascular endothelial cells at a ratio of 3:1. Endothelial cells were used ad supporting cells in hepatic differentiation. Differentiated cells in co-culture system exhibited higher hepatocyte-like function in a wide range compared to single human iPSCs cultured either in vitro or in vivo. Enzymes related to the detoxification, ammonia synthesis and the level of Alb and coagulation factors were higher using multicellular culture compared to the single-cell model [42]. All these findings point that the application of multicellular differentiation strategies could be a novel technique in the restoration of liver function.

## Tissue engineering strategy

Diabetes, heart failure, and hepatic failure are diseases of an enormous burden to the world, and current therapies for these often-lethal diseases are clearly inadequate. Tissue engineering holds great promise for the treatment of these diseases [43]. Millions of people currently suffer from the effects of chronic disease. Due to the limited availability of donors, only a fraction of individuals who could benefit from organ transplantations actually receive them. One possible avenue for remedying this situation is to artificially engineer human tissues [44–51]. One of the central themes of tissue engineering is to reproduce the body’s architectural and geometric intricacies, including vital cell–cell interactions. Tissue engineering techniques have been successfully applied to engineer many types of tissues; however, many challenges regarding their development still remain [44]. Numerous strategies have been developed to engineer tissues, with the most commonly used technique exploiting transplanted biofactors, such as cells, genes or proteins, into a porous degradable material called a scaffold. These scaffolds serve as synthetic ECM that organize the embedded cells into a 3D architecture and present them with stimuli for their growth and maturation. The ideal scaffold (1) contains properties that facilitate cell attachment, (2) contains a nutrient-rich environment to maintain cell viability and (3) biologically degrades overtime at a rate similar to the rate at which cells deposit matrix and proliferate in their new environment. One of the most commonly used approaches to tissue engineering is to seed cells on biodegradable scaffolds. These scaffolds bring cells in close proximity to each other and enable the formation of cell–cell contacts that mimic cells in the body. Ideally, the scaffold degrades at a rate similar to the rate of cell growth and matrix deposition, eventually completely replacing the synthetic scaffold with natural cells and matrix. However, challenges remain with this approach. For example, lack of vascularity in most tissue engineering constructs results in cell necrosis and loss of function, thus limiting the maximum size of tissue engineering constructs. For more detailed information on bioengineered tissue vascularization, readers may refer to the review paper of Kottamasu et al. [52]. In addition, difficulties in uniformly seeding cells throughout the various scaffolds prevent high initial cell seeding densities and finally the inability to mimic the complex cell–microenvironmental interactions, such as 3D orientation and architecture of cells, as well as homotypic and heterotypic cell–cell contact with microscale resolution [53].

## Three-dimensional tissue constructs

Over the years much has been attempted in generating hepatic tissue-engineered organs. One strategy for engineering 3D hepatic tissues is to cultivate cells within biodegradable scaffolds made from either natural [54, 55] or synthetic [56–58] materials. The scaffolds function as a 3D structure on which the hepatic tissue may be induced to grow. These scaffolds aim to mimic in vivo conditions and facilitate the delivery of nutrients, oxygen, and other factors [57–60]. The addition of specific microenvironmental factors can be used to enhance the function of these engineered organs. For example, by co-culturing non-parenchymal cells such as endothelial cells and fibroblasts, hepatocyte function can be prolonged [60]. A major challenge in liver tissue engineering is that the liver cells quickly lose their differentiated function. This is in contrast to the behaviors of hepatocytes in the body which have the capability to regenerate. Thus, it is desirable to formulate alternative approaches to more precisely control the organization of cells and vascularization of engineered hepatic tissues.

Traditional 3D scaffolding approaches are not suitable for generating such complex structures due to a lack of control of the tissue architecture and cell–cell interactions. In particular, hepatic cells in 2D culture as well as within traditional 3D scaffolds simply do not organize as they do in normal tissue; their metabolic properties are therefore unsuitable for liver tissue engineering applications. Hepatic cells or liver tissues are usually cultured in vitro in diagnostic applications or before implantation in therapeutic applications, where they interact directly within different natural or synthetic biomaterials or scaffolds for growth and functional liver maintenance. Biomaterials technology aims to make an advance in hepatic tissue engineering by developing the basis for fabricating tissues made from soft materials such as hydrogels with engineered microvasculature. Although engineering microscale features into tissue engineering scaffolds have been attempted before [53, 61–65], there are still several approaches that will eliminate the difficulties associated with other micro-fabricated tissue engineering scaffolds such as uniform cell-seeding.

## Hydrogels

Hydrogels have attracted great interest as scaffolding materials for tissue engineering because of their high water content, biocompatibility, and mechanical properties, which resemble those of natural tissues [66, 67]. Hydrogels have been used for tissue engineering of bone [68–70], cartilage [71–73], vascular [74] and other tissues [75, 76]. By adding cells to a hydrogel precursor prior to the gelling process, cells can be distributed homogeneously throughout the gel. In addition, hydrogels can be used to deliver soluble or immobilized signaling molecules to cells, act as support structures for cell growth and function, and provide space-filling for future tissue ingrowth [66, 67, 77, 78]. In general, hydrogels from natural sources can be derived from polymers such as collagen, HA, fibrin, alginate, agarose or chitosan [66]. Depending on their origin and composition, various natural polymers have specific utilities and properties. Many natural polymers, such as collagen, HA and fibrin, are derived from various components of the mammalian ECM. The advantages of natural polymers include low toxicity and biocompatibility. Collagen and other protein-based polymers are effective matrices for cellular growth, as they contain many cell signaling domains present in the in vivo ECM. Collagen gels can be naturally created without chemical modifications. However, in many cases, these gels are mechanically weak. To synthesize gels with enhanced mechanical properties various methods have been developed such as chemical crosslinking [79, 80], crosslinking with UV or temperature [79, 81], or in mixture with other polymeric agents [79, 82]. Hydrogels can be used to deliver soluble or immobilized signaling molecules to cells, act as support structures for cell growth and function, and provide space-filling for future tissue ingrowth [66, 67, 77, 78]. For example, growth factors, such as TGF-β have been tethered to PEG hydrogels to regulate smooth muscle cell function [83] and BMP-2 has been covalently attached to alginate to regulate osteoblast migration and calcification into the gels [84]. Also, differentiated cardiac tissues have been engineered by casting neonatal rat cardiac myocytes into collagen gels and subsequently subjecting them to cyclic mechanical stretch [85]. Another type of hydrogels has been used extensively in tissue engineering approaches [47, 86–101]. The most abundant heteropolysaccharides in the body are the GAGs. They are long unbranched polysaccharides containing a repeating disaccharide unit that contains either of two modified sugars: *N*-acetylgalactosamine or *N*-acetylglucosamine and a uronic acid such as glucuronate or iduronate. GAGs are located primarily on the surface of cells or in the ECM. HA is a GAG that is particularly prevalent during wound healing and in joints. Covalently crosslinked HA hydrogels can be formed by multiple chemical modification means [102–105]. HA can be degraded by cells through the release of enzymes such as hyaluronidase. HA is particularly appealing for tissue engineering as it is naturally present in great abundance in a variety of tissues [106–108]. Previously, HA scaffolds have been used for tissue engineering of various tissues [55, 104, 109]. In addition, composite HA-PEG scaffolds have been used for tissue engineering [109–111]. Specifically designed amphiphilic peptides that contain a carbon alkyl tail and several other functional peptide regions have been synthesized and shown to form nanofibers through self-assembly by mixing cell suspensions in media with dilute aqueous solutions of the peptide amphiphile. Nanoscaled fibers produced by self-assembly of peptide amphiphile have great potential in tissue engineering [112, 113]. Peptide groups may be customized to direct cell behavior and polymerized directly into the hydrogel. For example, it was shown that directed differentiation of neural stem cells could be modulated using such a hydrogel functionalized with isoleucine–lysine–valine–alanine–valine, a laminin-derived sequence, without the use of additional biochemical factors [114]. Despite their excellent biological properties and the ease with which they can be modified for specific applications they are mechanically weak, which has limited their application in vivo.

Along with chemical properties, physical parameters of fabricated hydrogels such as stiffness, elastic modulus, degradation, and ligand density have a profound impact on stem cell fate decision and phenotype acquisition. Stiffness and matrix composition together have potential to regulate the hepatic differentiation of progenitor cells [115]. In a very recent experiment, cellular reaction of stem cells to matrix stiffness was evaluated using acrylamide and bisacrylamide in a variable stiffness. It was found that stem cells cultured on a soft substrate tended to significantly acquire hepatocyte-like cell phenotype on a soft matrix compared to cells of stiff matrix system. Additionally, soft matrix can maintain the function of stem cells longer than stiff matrix [116]. The soft matrix actively promoted cell migration and alignment. Young’s modulus value and integrin β1 content were decreased by increasing matrix stiffness [117]. These results give deep insights to cell biologists in designing improved matrices with abilities to enhance differentiation efficiency. To our knowledge, there are several mechanistic studies evaluating the effect of microenvironment biophysical properties on hepatic differentiation and functional behavior but more investigations are highly demanded to discover beyond molecular mechanisms.

## Hepatic differentiation of various stem cells on decellularized scaffolds

Decellularized liver scaffolds are achieved by isolating of all cellular and immunogenic components of extracellular matrix proteins used commonly in studies related to liver regeneration. The ECM can be obtained through various methods including chemical, mechanical and enzymatic alone or in combination with each other aimed to preserve maximum ECM composition and natural architecture with minimum damage [118, 119]. Evidence points out that the preserved components of ECM differ in number and type depending on the method of decellularization. While in a method the greater amount of components is preserved, however, the substrates required to support hepatic cells or tissue carrying stem cells such as fibronectin, collagen and GAGs may be also excluded [120]. Experiments showed endogenous cytokines and growth factors attached to side chains of GAGS either can be conserved after decellularization [17]. In a very recent study, mass spectrometric analysis revealed that the amount of ECM proteins in a liver decellularized by deoxycholate were significantly higher than those decellularized by ammonium hydroxide, but the retained ECM proteins in the latter mainly were fibronectin, collagen (types 1 and 4) and proteoglycans, participating in cell attachment, survival, growth and hepatic differentiation [120]. Since the type and amount of the material content of scaffold affect the orientation of stem cells toward the hepatic lineage, quantitative and qualitative evaluation of decellularized scaffolds component appears to be essential prior to recellularization [120]. ECM scaffolds in the field of liver tissue regeneration are originated from various sources. For instance, the whole or part of the human liver organ which is not suitable for transplantation or the addition of synthetic or semisynthetic bio-molecules is at the center of attention [121].

Considering the role of common signals in hepatic differentiation across the various species, xenogenic animal livers including porcine, rat and mice also are used [122]. It seems that hepatic cells could per se trigger the stem cell-to-hepatocyte differentiation by providing essential ECM in in vitro condition. Liver-derived cell lines such as HepG2, Sk-Hep-1, liver primary cells, and progenitor cells were shown to release the array of ECM components into the culture media, promoting the hepatic commitment of various stem cells. Although decellularized whole liver maintains as much of native liver architecture, it requires challenging perfusion systems to fulfill decellularization which is not suitable to study a certain area of tissue [123]. Decellularized liver scaffolds not only markedly up-regulate the gene expression of integrin receptors, α_1_, α_2_ and notably α_3_β_1_ subunits, and related downstream modulators such as FAK and ILK but also yields the induction of HGF, bFGF, oncostatin-M and MAPK genes. Using scaffolds with higher porcine liver ECM content, hepatic differentiation of bone marrow-MSCs is not accelerated in optimum rate and expression of hepatic markers disappeared [124].

Though it is well-established that the ECM composition plays an important role in the determination of cell attachment, differentiation, and proliferation, however, a great body of studies has particular attention on the induction and/or inhibition of signaling pathways by growth factor treatment [125, 126]. Notably, there is little knowledge regarding the role of ECM type, protein content, and protein types of the scaffold in hepatic phenotype acquisition. As a matter of fact, novel strategic approaches investigating these features seem to be highly recommended. The use of plates coated with Matrigel, laminin and different types of collagen with a limit number of adhesion molecules was done previously. It has been shown that these matrices were unable to support the attachment of hiPSC-derived definitive endoderm cells [121]. In contrast, DE cells not only attached more efficiently to plates containing HepaRG acellular matrix and tightly adhered to each other but also committed more efficiently toward hepatic lineage [123, 127]. The effect of matrixes was examined on later stages of hepatic differentiation and shown that matrices mostly induce mature hepatic markers expression when used at maturation step [14]. On the other hand, the use of the human decellularized liver matrix in the early stage of differentiation can further stimulate earlier high expression of stage-specific markers in hepatic differentiation of hiPSCs compared to cells grown on Matrigel and collagen substrates [121]. With the progress of hepatic differentiation, the cells lose their sensitivity to the ECM composition. Therefore, ECM composition has the least influence on hepatic differentiation at the late stages. If the entire process of differentiation takes place on liver decellularized matrix, the large number of cells further display hepatic lineage features and the pattern of markers expression is found in embryonic development of liver and a low expression of HLF and HHEX, as mature liver markers, indicates these cells do not enter to a complete maturation procedure [121]. However, evaluation of ECM effect on the maturation of hepatocytes-derived from iPSCs led to achieve cells closer to intact mature liver cells evidenced by expression of lower levels of fetal liver markers, AFP and CYP3A7, and greater extent expression of Cyp proteins as well as better metabolic activity as compared with the same cells cultured on synthetic 3D scaffolds [128].

Some alternatives have been represented to partially overcome immaturity including pretreatment of cells with divalent cations such as Mn^2+^, as an integrin activator, remove of HGF, and the addition of AMP and Matrigel in the maturation step of hepatic differentiation [123, 124]. One recent study has shown that liver progenitors seeded onto ECM discs developed from ferret rat decellularized liver can self-assemble into 3D spheroids with a greater capacity for differentiation into hepatocyte and simultaneous generation of bile canaliculi-like structures [122]. The cultured cells also showed the first step of xenobiotic catabolic activity and expression of adult livers markers (HNF4a, CYP3A4, and CK19) [122].

In another study, the hepatic induction of porcine-iPSCs in a mixture of porcine liver ECM solution and hepatocyte differentiation media (10% of ECM in a total volume of media produced mature and functional cells compared to 2D cultures [14]. Following the transplantation of these cells to decellularized rat liver, the cells establish a cell-to-cell connection and efficiently attach to the matrix and exhibited enhanced expression of AFP and Alb, indicating hepatic phenotype maintaining [14]. The differentiated cells showed morphology similar to hepatocytes, abundant cytoplasm and large microvilli with a high ratio of cytoplasm to the nucleus. The transplantation of hepatocyte-like cells restored the liver function and reduced the CCL4-induced liver fibrosis in mice when compared to non-ECM cultures [14]. Despite considerable advances in liver tissue engineering, the use of liver decellularized scaffolds encounters some obstacles [129]. The problematic issues in the liver are that the outcome of decellularization show donor to donor variation [129]. Indeed, using the same chemical decellularization protocol on five livers obtained from patients undergoing resection and three healthy porcine liver yielded five different outcomes for human livers but reproducible for porcine livers [129]. In addition, substantial leakage of cells from the vascular network and parenchymal space takes place to the outside of the scaffold in perfusion-based protocols [130]. Based on the size of scaffolds, appropriate methodologies must use for decellularization. For example, the perfusion of small-size livers is laborious and many tissue masses are required to yield tissue powder. However, a low number of large livers are needed for the preparation of tissue powder while comparable to the human liver [14, 131]. In addition, the vacant spaces of tissue undergo collapse after removal of cells, which in turn makes the tissue condensed and stiff. Obtained stiffness is similar to the consistency of fibrotic tissues such as liver parenchyma. It has been reported that tissue stiffness could be altered from 4.5 kPa in normal tissue to more than 19 kPa in diseased liver tissue. Stem cells differentiated toward hepatic lineage on soft matrices (stiffness = 0.4 kPa) demonstrated a high degree of hepatocyte characteristic within a few hours, whereas stiffer matrices (stiffness > 80 kPa) was unable to support differentiation. Integrins have been suggested play an important role in hepatic induction of stem cells and the matrices with moderate stiffness enhance cell–cell (catenin-based) and cell–matrix (integrin-based) adhesion through maintaining balanced regulation of integrin-β1 and β-catenin expression and exhibit high value of Young’s modulus [117].

The ultimate goal of liver decellularization is to subject bioscaffolds for recellularization which are more applicable to hepatic microstructure. Liver decellularization and recellularization reported by Uygun et al. was the first study in this area. They transplanted acellular scaffolds to hepatic tissue coincided with the administration of 5 × 10^6^ rat primary cells through the portal vein. Evaluations confirmed the successful engraftment exceeding 90% percent. During the 1st days after recellularization, transplanted cells surrounded the large veins and distributed across the whole scaffold. Based on the released data, approximately 20% of transplanted cells exhibited apoptotic changes. Furthermore, the cells continuously expressed the UDP-glucurotransferase 1, polypeptide A1, Alb, and urea. Interestingly, the expression of cytochrome 450 isoenzymes was similar to the pattern observed in normal liver. Also, Robertson and colleagues examined hepatic recellularization by using adult rat and human hepatocytes in a bioreactor culture model. After perfusion of rat liver with rat or human hepatocytes through the portal vein, cellular constructs were developed after 28 days and reticular networks consisted of type III collagen III formation were observed. In addition, these cells were functional and actively showed Alb and urea synthesis for up to 28 days of study [132]. In another study, decellularized mouse liver was recellularized with 5 × 10^5^ iPSCs-derived hepatocytes and transplanted into the mice by a four steps infusion protocol through the portal vein. The transplanted cells successfully engrafted around the portal vein and produced Alb and α-fetoprotein [14]. It was also shown that decellularized hepatic scaffolds were appropriately recellularized in rat partial hepatectomy model [133]. Although results from recellularization protocols seem promising many efforts are essential to achieve functional liver tissue. Calling attention, the various routes of recellularization demonstrated different performance and regenerative outcomes. In a comparative experiment, the efficiency of cell administration was assessed via portal vein and bile duct routes in murine recellularized liver tissue. It was shown that administrated cells developed aggregates that block the microvasculature system and led to uneven distribution of cells throughout the parenchyma when cells were introduced through the portal vein. In contrast, by cell transplantation through bile duct, these cells were uniformly distributed throughout decellularized hepatic parenchyma [134]. Some authorities declared the potency of decellularized non-hepatic bioscaffolds such as placental tissue for the restoration of hepatic function [135]. Despite these technical barriers, considerable efforts are ongoing to generate liver decellularized scaffolds closely simulate in vivo microarchitecture of the liver.

## Hepatic differentiation via the combination of cell source and nanotechnology-based approaches

The sophisticated nanofiber designed is touted as alternative methodologies for improving the efficiency of fabricated biomaterials in the favor of target tissue regeneration. The electrospun nanofibers with high surface area and porosity have been extensively used to act as backbone ECM, improving the growth and differentiation of cells. For example, the 3D liver model developed by electrospun chitosan nanofibers and co-culture system was shown to support hepatocytes and fibroblasts for long-term liver functions. The use of different ECM substrates in synthesized nanofibers such as fibronectin enhanced cellular adhesion and polarization [136]. Chitosan is a suitable polymer for hepatocyte culture, expansion and maintenance, because of structural similarity to glycosaminoglycans; the components seen commonly in hepatic ECM [137]. Based on the great body of documents, hepatocytes can maintain their morphologies and functions for long periods of time in the co-cultures 3-D system [136]. By applying various strategies, it is mighty to preserve the functional behavior of the target cells in a controlled manner. For instance, encapsulation of hepatocytes in 3D structures provides an appropriate niche for optimal function. In one study, polyelectrolyte complex hydrogel fibers were used as self-assembling 3D structures to encapsulate hepatocytes and endothelial cells differentiated from human iPSCs. The co-culture of cells in multi-interfacial polyelectrolyte complex fibers with chitin and sodium alginate origin showed that the existence of endothelial cells in the scaffold significantly improved hepatocyte function and in vivo studies revealed the superior effect of endothelial cells in vascularization of the transplanted scaffolds [138].

Natural ECM has a nanoscale structure that is common to many tissues’ basement membranes. To mimic liver ECM, various nanomaterial-based scaffolds have been studied until yet. CNTs showing controlled nanoscale topography that can be employed as liver ECM [139]. In a study, PA was used as a base matrix to resemble the human liver due to its Young’s modulus [140]. In addition to the existence of anchor sites stimulating cell attachment, scaffold consistency and pattern could dictate specific phenotype for target cells. It has been shown that the scaffold stiffness can influence the in vitro behaviors of hepatocytes [141]. Also, PEG-CNTs were coated on the PA to make CNT-PA. The influence of CNT-PA on the differentiation of hAECs to functional HLCs was investigated. CNT-PA had the potency to induce the up-regulation of hepatic markers at transcription and protein levels. Moreover, this milieu was able to yield higher uptake of indocyanine green, Alb secretion and comparable CYP3A4 enzymatic function [19].

Authorities demonstrated broad advantages for tissue engineering governed by CNTs. For example, they are biocompatible materials with great potential as cell-supporting substrates, provide strong mechanical properties, can be easily functionalized and are aligned as the collagen fibers [142]. Calling attention, MWNTs used as a scaffold to investigate the effect of it on primary liver cell culture showed suitable adhesion, proliferation rate with an enhanced function. This microstructure possesses the capacity to force hepatocyte to enhance Alb production coincided with the activity of CYP1A2 enzyme [139].

Hepatocyte growth factor has been found to play an important role in morphogenesis, and liver regeneration. Because of its short half-life in circulation, specific carriers have been studied for improving the release profile [143]. The polymeric delivery systems based on PLA, PLGA, and polycaprolactone and inorganic nanomaterials such as silica nanoparticles have been investigated. Due to specific properties such as biocompatibility, easily functionalized surfaces and tunable pore volumes silica nanoparticles are considered in the field of tissue engineering [144]. The modification of MSNs supplemented with PEI and loaded with Activin A, aFGF and HGF were used for hepatocyte differentiation of mouse embryonic stem cells. Functionalized MSNs were found to deliver growth factors in a sustained manner. Compared to the control non-treated cells, the gene expression of hepatocyte markers, such as ALB and alpha-fetoprotein was upregulated in the GF–PEI–MSN complexes. Also, the expression of hepatic functional markers such as α_1_-antitrypsin, cytochrome P450 subunit CYP7A1, and glucose-6-phosphatase were higher in GF–PEI–MSN complexes. Monitoring the synthesis of glycosaminoglycans by periodic acid-Schiff showed that the increasing number of positive cells in the GF–PEI–MSN complex group. In the presence of this nanoparticle, mouse embryonic stem cells acquire a potency to give rise to endodermal lineage and hepatocyte-like phenotype after transplantation into the injured liver in the mouse model [145]. In conclusion, nanomaterials possess advantages for tissue regeneration. For example, the nanofibers act as ECM and provide high surface area and porosity that are suitable for cell proliferation and differentiation. CNTs simulate collagen fibers and due to their high mechanical properties provide good stiffness as a scaffold. Also, nanomaterials can be used as carriers for improving release completeness of growth factors that have a role in the differentiation of cells. It suggests that the combination of these nanomaterials can be appropriate for hepatic differentiation.

## 3D printing role on cell fate toward a hepatic-like phenotype

3D printing has been touted as a novel method to dictate specific cell alignment on distinct substrates. During the last decades, many attempts have been collected to commercialize 3D-printing scaffolds. In this regard, Organovo™, one of the pioneer bioprinting corporations, has effectively attained 3D-vascularized liver constructs with high cell survival rate and applicable microstructures coincided with the formation of hepatic lobules after the use of mixed cell populations notably hepatocytes, endothelial, and hepatic progenitor cells [146, 147]. As a matter of fact, 3D tissue-engineering technology possesses the capacity to effectively simulate complex organ formation in a 3D pattern and circumvent plenty of obstacles reported for the 2D culture system [23]. The main obstacle is a confined hepatocyte proliferation and expansion rate in the 2D system. For instance, the hepatocyte proliferation rate is commonly decreased 3 to 5 days post-isolation and these cells are prone to de-differentiate and acquire fibroblast-like phenotype by modulation of hepatic-associated genes, leading to cell functionality removal and limitation of activity [148, 149]. It has shown that the secretion of Alb, synthesis of urea, and expression of gene *CYP3A4* are decreased in freshly isolated hepatocytes [150, 151]. In support of the above-mentioned statement, the fabrication of scaffolds with natural substrates enabling a long-term culture of hepatocytes is the most attractive issues remain to be resolved. Investigations of primary hepatocytes in the 3D niche have been briefly progressed to elucidate hepatic metabolism and uptake [150, 152]. Calling attention, primary hepatocytes have the potential to form individual micro-aggregates in organ culture systems [23]. Culturing in 3D alginate scaffolds was found to promote hepatic cells aggregation 7 days post cultivation. The cells acquired prerequisite function and typical hepatocyte-like morphology to synthesize Alb [24, 151]. Considering the importance of cell aggregation in the function of hepatocytes, this leads to the expansion of a diversity of techniques to encourage and dictate the formation and maintenance of aggregates and micro-spheroids within an engineering scaffold prior to implantation to the target sites in vivo [153, 154]. In line with this claim, encapsulation or loading single-cell suspensions onto a porous scaffold are famous techniques to induce cell aggregation [155, 156]. However, some considerations in scaffold design are highly recommended for appropriate results. For example, the control of aggregate size to avoid generation of necrotic cells located at the core or applying some approaches to induce reciprocal interconnectivity between cells from neighboring aggregates while facilitating nutrient diffusion and neo-vascularization following implantation [157]. Notably, some inherent modulations could not be omitted during the fabrication of synthetic scaffolds. In scaffold production, restriction and bottlenecks such as freeze casting, electrospinning, salt leaching, or gas foaming could change the size and cell microspheres integrity within 3D backbones [158]. In these circumstances, only a limited degree of control is possible over scaffold geometry, pore size, and interconnectivity.

Hexagonal lobule-like geometries have been displayed to have advantageous effects on cultured hepatocytes [118, 159]. Use of an engineered technique to re-form detailed lobule structure must be considered as lobule organization in vivo is recognized to have a biological effect on blood and bile flow as well as hepatocyte phenotype and zonality [160]. The influence of 3D-printed scaffold pore geometry has therefore not been thoroughly examined for applications in soft tissue engineering. This may partially be due to the complications in 3D printing of soft resources, mainly hydrogels, making laborious attempts. Recently Sainz and co-workers studied the effect of gelatin-based scaffold pore geometry on non-adherent cells while keeping pore size constant and compared the result with a control 2D surface. They revealed that 3D manipulation of quite simple material, such as gelatin, can have major influences on the application of engineered tissues [161]. Compared to the 2D system, an appropriate phenotype acquisition and functional improvements are seen in 3D printing systems. In 3D hydrogel-based culture model, the co-culture of hiPSCs and hematopoietic progenitor cells with endothelial lineage and adipose-derived stem cells contributed to microscale size hexagonal construction while the biochemical activity of hepatocyte was initiated by advanced liver-specific gene expression levels, increased cytochrome P450 stimulation and activated secretome [118].

### Bioinks

Hydrogel bioinks used for cell-based systems presented biochemical and biophysical motivations to mimic native ECM in in vivo microenvironment. Bioinks with the ability to allow appropriate cellular activities have also been improved for 3D bioprinting. In this technique, interfacing between the substrates and fabrication hardware, physicochemical features of biomaterials and bioink cocktails are one of the most obstacles attendant with 3D bio-fabrication methods [162]. Different studies have used a diversity of hydrogels such as HA, MeHA, PEG, GelMA, NovoGel^®^ 2.0 (a non-adherent and thermo-responsive hydrogel), collagen, fibrin, fibrinogen, PCL, gelatin, and alginate in different combinations, for 3D bioprinting of hepatocytes and liver associated cell lines [118, 162–165]. Based on previously published experiments, different bioink formulations were used for the rapid production of hepatic tissue. Two forms of cell-loaded scaffolds including hepatic cells, gelatin and/or fibrinogen were effectively synthesized by programmed rapid prototyping method and became stable with thrombin. During the procedure, no obvious cell damage was observed. It was reported that an equal gelatin/fibrin combination provided the highest mechanical properties. Hepatic cells were demonstrated to efficiently proliferate in the matrix of gelatin/fibrinogen. The Alb biosynthesis was found to improve in embedded hepatocytes. It seems that fibrin performs as a satisfactory material for a gelatin-based cell assembly matrix with easy manipulation, bio-resorbable, supporting in vitro cell functions [166]. Another group combined ADSCs within a gelatin/alginate/fibrinogen hydrogel to construct a vascular-like network, using a digital pattern. They also located a cocktail of hepatocytes with gelatin/alginate/chitosan around it to simulate a natural liver tissue. In these systems, endothelial growth factors were used to induce the ADSCs to trans-differentiate into endothelial-like cells. In these bulk constructions, a 3D syringe-based bioprinting technique is demanded to provide bulk interchange channels using to preserve the integrity of the constructed liver structure. This double-nozzle assembling techniques have the potential to be a powerful apparatus for producing multipart constructs with distinctive intrinsic/extrinsic properties [167].

### 3D fabrication procedure

Recently, several 3D bioprinting technologies have been exploited with effective advances in shaping biomaterials at the macroscale size to design biomaterials with high complexity. These technologies can be divided into different categories that include stereolithography, laser-assisted forward transfer, nozzle-based bioprinting techniques, shear-thinning extrusion bioprinting, sacrificial bioprinting, microfluidic bioprinting, and multi-material bioprinting [168, 169]. Depending on the type of bioinks, some of these methods are applicable for liver tissue engineering.

### Shear-thinning extrusion bioprinting

Extrusion bioprinting is a promising method to produce organized tissue constructs that supply cells and matrix materials concurrently to restore or substitute damaged or diseased tissues and organs. This procedure has an interesting vision to fabricate constructs with 3-D distinctions of cells through numerous axes with great architectural complexity. Extrusion bioprinting naturally needs the distinct bioink formula to be self-supportive and preserve the architectural integrity of the scaffold upon extrusion [170]. Lee et al. produced a GelMA-based shear-thinning bioink that allowed direct extrusion, by rapidly cooling down GelMA solutions to beneath their gelation points in the printing nozzle [170]. UV-initiated crosslinking could then be used to subsequent solidification [170, 171]. The bio-printed products could maintain the viability of encapsulated hepatocellular carcinoma cells; HepG2 and mesenchymal stem cells (MSCs) within the GelMA microfibers over 14 days [170].

### Multi-material bioprinting

Printing a single bioink has been the base of most researches in 3D-bioprinting customarily. Nevertheless, because of the complexity of organs and tissues, combining various construction biomaterials and numerous cell types is essential in a single bioprinting procedure. Several studies have been done to explore the intricate associations between the printing factors and the resultant resolution. Tissue constructs made of numerous materials in a well-ordered fashion could be gained by serially altering the input bioinks for the digital micro-mirror device bioprinter [118, 172]. For instance, to simulate the liver microstructure, Ma et al. used the sequential printing of human hiPSC-derived hepatic cells in a hexagonal lobule structure with supporting cells from the endothelial and mesenchymal original, filling the lining of the lobules [118]. The 3D printed liver construct proved structural similarity with it’s in vivo counterpart and showed an appropriate functionality evident by prolonged cell viability, cytochromes activity, and stable secretion of liver biomarkers.

### DCW system

DCW is used to fabricate simple linear or complex conformal constructions on a substrate by material deposition. Digital writing or digital printing is a group of malleable multifaceted procedures in which different methods and devices such as inkjet, laser, mechanical pressure, and tips are used to create structures in the range of nm to the mm. Also, an extremely wide range of materials from ceramics, metals, polymers, and dielectrics to bio-materials is used in these techniques [173]. In new technical systems, solid freeform fabrication, cell-based micro-chip systems, sensors, and microfluidic devices, cells are combining as part of the construction blocks for several tissue engineering procedures. Multilayer 3D printing techniques combined with perfusion culture systems are capable of producing in vivo liver tissue. To produce a biomimetic liver micro-organ as a drug screening approach, a DCW method in combination with perfusion culture has been established [174]. To align alginate hydrogels encapsulating HepG2 cells, the DCW system with four nozzles capable of operating in extrusion or droplet model was utilized. Three-layer tissue-engineered scaffolds were created by printing these alginate-encapsulated cells into liver sinusoidal shape structure that combined into a microchip device, allowing for medium exchange. Outcomes presented that over 80% of HepG2 cells in the cross-linked, printed products stayed alive after 3 days. Additionally, viability was preserved after 24-h perfusion circulation, indicating that the perfusion method did not per se influence cell viability and could be utilized to perfuse a targeted drug through fabricates and measure pharmacokinetic performance. Another investigation successfully utilized the DCW process and incorporated both hepatic and epithelial cells in the Matrigel substrate to more precisely stimulated hepatic sinusoid hierarchical architecture. A microfluidic environment was designed by these cells to make enhanced higher reliability in the favor of micro-liver tissue stimulation. This application is useful to serve as a portable ground model for the study of drug conversion and radiation protection of living liver tissue analogs. It seems that understanding the underlying mechanisms of the multi-cellular biological system responses for a prolonged period, various disease models and biosensors need to be elucidated [175].

### Stereolithography

Stereolithography is one of the first 3D printing methods in which a forerunner mixture of a UV-curable material is filled in a tank, which is then cross-linked in an aligned manner. After the depositing of each distinct layer, a mechanized stage moves the deposited construction from the light source in the z-direction so there will be space for the subsequent print layer [169]. Scaffolds that more closely simulate liver construction and encourage improved hepatocyte culture have also been considered yet. PEG-based, photo-polymerizable polyethylene glycol-based hydrogels have been produced using stereolithography to develop hepatocyte cell seeding, proliferation, and duration of perfusion cultures in liver designs. Hydrogel scaffolds with open channels were fabricated for post-seeding and perfused culture of primary hepatocytes that form 3D structures in a bioreactor. Cell seeding densities, flow rates photo-initiator concentrations, stereolithography energy dose and pretreatment conditions were determined to be important factors to control and maximize cell viability. The perfused culture of primary hepatocytes in hydrogel constructs in the existence of soluble epidermal growth factor increased the preservation of Alb creation throughout the 7-day culture relative to 2D controls. This method can be hired to construct soft scaffolds for a number of bioreactor configurations [176].

### Other physical factors important in the efficiency of bioink

Different studies have shown that cell viability is affected by several factors including the material flow rate, material concentration, dispensing pressure, and nozzle geometry. These results can attend as an instruction for future explorations and optimization of the 3D system. Sun and his co-workers made a multi-nozzle bio-printing system to concurrently deposit cells and multiple biomaterials. Their rheology study and cell viability assay were executed to examine mechanical-stress-induced cell damage during the printing procedure [177, 178]. Both Burdick and Wells groups examined how the differentiation of HSCs was affected by matrix stiffness. Tissue fibrosis that might be in part attributed to ECM stiffening during disease improvement has been the cause of differentiation of HSCs into myofibroblasts. Their outcomes established that HSCs cultured on stiffer hydrogels (24 kPa in elasticity) as compared to HSCs on softer hydrogels (2 kPa), expressed advanced levels of alpha-smooth muscle actin and type I collagen [179–181]. Shear stress applied during the printing procedure is another factor that can affect cell viability. Using liver spheroids can protect the cells from the adverse influences applied by the shear stress during the printing process and recapitulate the cell-to-cell interactions. By their stable secretion of hepatic biomarkers including Alb, ceruloplasmin, alpha-1 antitrypsin, and transferrin, the bio-printed liver spheroids surrounded by GelMA hydrogel showed long-term functionality for up to 30 days [163]. In another experiment, it was revealed that UV exposure can affect cell viability in UV-initiated crosslinking systems which are used to subsequent solidification of self-standing multilayered constructions and thereby advanced UV exposure could improve the crosslink density and thus causes in decreased cell viability [170].

## Hepatic phenotype acquisition via an intracellular signaling pathway

### Small molecule

To induce hepatocyte-differentiation of stem cells, different methodologies have been developed yet. In most circumstances, exploiting step-wise differentiation approaches along with recombinant growth factors or small-molecule analogs could be promising [182]. Early protocols for the generation of the specific cell type from stem cells was applicable by using embryoid body formation [183]. The basis of this protocol was the spontaneous formation of cell aggregates (embryoid bodies) from pluripotent cells which has the capacity to give rise to all three germ layers [16]. To expand the hepatocytes, the most well-organized method is a monolayer culture of distinct cells in a step-wise manner exposure to specific cocktails of recombinant growth factors and cytokines, including Activin A, Wnt3a, HGF, OSM, FGF4, VEGF, EGF and BMP4 [184–191]. These approaches were designed to simulate the embryonic stages of hepatocyte lineage. The protocol has, however, some deficiencies with low differentiation efficacy and heterogeneity in the nature of differentiating cells [182].

Small molecules can regulate specific target(s) in signaling pathways and epigenetic mechanisms have been recognized as valid chemical tools for mediating cell fate. The use of small-molecule compounds has the advantage of being safer than the use of cytokines, nucleic acids, or protein therapies [192]. Other advantages of small-molecule-based hepatic differentiation protocol in comparison with other methods are simplicity, stability, reproducibility, and low cost [193]. Thus, the identification of small molecule compounds has enhanced the development of stem cells to liver regeneration. In the following, we will discuss the role of small molecules differentiation of stem cells toward liver cells in various studies. During hepatic differentiation of human MSCs, Wnt/b-catenin signaling is inhibited. Inhibition of Wnt/b-catenin signaling molecules or target genes induces the hepatic differentiation of human MSCs. Effective modulation of Wnt signaling and glycogen synthase kinase 3 inhibition using small molecules efficiently produced definitive endodermal lineage. Dickkopf Wnt signaling pathway inhibitor 1, an antagonistic inhibitor of the Wnt signaling pathway was shown to promote hepatic differentiation of bone marrow-derived MSCs [33, 194]. More recently, different research groups showed that glycogen synthase kinase 3 inhibition with CHIR99021 efficiently directs hPSCs toward the definitive endodermal lineage and primary hepatocytes, via a combination of small-molecule treatments with dimethyl sulfoxide. They stated that the small-molecule based approach is a simple, inexpensive and reproducible platform for the derivation of hepatocytes from hPSCs [182, 195]. Itaba et al. screened 23 newly synthesized derivatives of small molecule combinations produced from several known Wnt/β-catenin signal inhibitors. In their study, HC-1, IC-2, and PN-3-13 similarly suppressed Wnt/b-catenin signaling, however, IC-2 was the most effective inducer of hepatic differentiation. Interacted molecules with IC-2 were not determined in their studies. IC-2 is a derivative of ICG-001. It has been made clear that ICG-001 suppresses Wnt/b-catenin signals by binding CREB binding protein, and because IC-2 is derived from the ICG-001, it may act with the same mechanism as the ICG-001 [196].

Stauprimide a small molecule described by Shoutian Zhu et al. that can significantly increase the efficiency of directed ESC differentiation in conjunction with lineage specification cues. Using an affinity-based approach, NME2 was reported as the biological objective of stauprimide [197]. Inhibition of NME2 occurs when stauprimide binding to NME2, NME2 nuclear localization, which, in turn, suppresses c-Myc expression. Downregulation of c-Myc expression, a key factor in the preservation of ESC self-renewal and its inhibition facilitates the process of differentiation [198].

Tasnim and co-workers stated a protocol for efficient differentiation of hESCs into hepatocyte-like lineage using a mainly small molecule-based approach. These three stepwise differentiation strategies contain the use of optimized concentrations of LY294002 and bromo-indirubin-3′-oxime) for the generation of definitive endoderm; sodium butyrate and dimethyl sulfoxide for the generation of hepatoblasts and SB431542 for differentiation into hepatocyte-like cells. They also showed that SM-Hep were morphologically and functionally similar or applicable to the hepatocytes generated by growth factor cocktails (GF-Hep) in terms of expression of hepatic markers, urea and Alb production and cytochrome P450 (CYP1A2 and CYP3A4) activities [33]. Ouyang et al. introduced 2-(4-bromophenyl)-*N*-(4-fluorophenyl)-3-propyl-3H-imidazo [4,5-b] pyridin-5-amine (SJA710-6), as a small molecule able to selectively promote MSCs differentiation toward hepatocyte-like cells after monitoring a library of 2500 small molecules [199]. Also, small molecules of RNA called small hairpin (shRNA/Hairpin Vector) can be used as another small molecule to increase the regenerative capacity of hepatocytes. By small hairpin RNAs (shRNAs) directly in the animal model, Wuestefeld et al. recognized the MKK4 which is dual-specific kinase as a key mediator of liver regeneration. MKK4 inhibition expressively amplified the regenerative ability of hepatocytes in animal models of liver regeneration. Notably, MKK4 inhibition in hepatocytes decreased fibrosis after chronic liver injury. Hepatocytes with stable RNA interference (RNAi)—mediated MKK4 silencing indicated quicker cell-cycle entrance and accelerated liver regeneration [200].

In summary, using small molecules instead of growth factors would provide a striking alternative source since small molecules are cell-permeable and non-expensive compared to growth factors [201]. Due to several advantages including the ability for sequential, tunable and sectional control of specific protein function, elimination of the risks and drawbacks associated with genetic manipulation, the low efficiency and slow kinetics of distinct phenotype induction, the application of small molecules in the biomedical field could simplify therapeutic benefits related to stem cells [201, 202].

## miRNAs

As above-mentioned many studies have tried to discover cheap and accessible techniques for producing definite liver cells or hepatocyte-like cells from different stem cell types. In order to achieve this end, several hepatic differentiation media with different growth factors and cytokines have been introduced to hinder hADSCs-differentiation into hepatocytes. In a recent experiment, the significance of specific miRNAs has been shown on the differentiation and growth of certain cells. microRNAs are noncoding RNAs that control gene activities at the post-transcriptional stage, regulating stem cell biological features in plants and animals [203]. Further, 2500 unique mature human miRNAs have been recognized until now and it is probable that more than one-third of human protein-coding genes are exposed to regulation by miRNAs during the development of hepatic tissue. For example, the essential role of miR-30a has been recognized for bile duct development in the model of zebrafish [204]. The most abundant miRNA in the liver, miR-122, accounts for approximately 70% of total miRNAs [205], which is essential for the appropriate progression of hepatocyte differentiation [205, 206]. In recent years, evidence showed miRNAs performance in stem cell maintenance, differentiation, and organ development [206]. Data suggest miRNAs can be important regulators of hepatocytic differentiation. Additionally, studies showed that miR-194 convinces intestinal epithelial cell differentiation as well [207] is a key moderator of chondrogenic differentiation of ADMSCs [208]. More recently, Jung et al. [27] showed that the amplification of miR-194 progressively increased in HepaRG liver progenitor cells and hESCs differentiation into hepatocytes. In a better word, miR-194 overexpression results in down-regulation of 16 genes in both HepaRG cells and hESCs by modulation of IGF1R and YAP1, a downstream mediator of Hippo signaling that plays a key role in cell fate determination [209]. It was elucidated that Let-7f miRNA acts as a negative regulator for hepatic differentiation in hADSCs through the suppression of HNF4a and silencing of Let-7f, promoting hepatic differentiation [210]. It seems that simultaneous modulation of different miRNAs, miR-122, miR-148a, miR-424, miR-542-5p and miR-1246, could stimulate hMSCs-HLCs transition. The HLCs originated from five miRNAs expressed hepatocyte-specific genes HNF4A, AFP, ALB, TF, CYP3A4, and G6P [211]. Möbus et al. found that miR-199a-5p repression increased hepatic differentiation led to the generation of hepatic cells. Further, they showed that these hepatic cells have the ability of engrafting and regeneration the mouse liver [28]. They recognized the ATPase subunit of the mammalian SWI/SNF complex, SMARCA4, as a target of miR-199a-5p. This complex consists of 15 subunits and participates in is chromatin-remodeling, used in iPS reprogramming and chromosomal stability [212]. These studies show that one or several definite microRNAs can be used to transform stem-cells derived from sources into hepatocytes to proficiently obtain hepatocytes in vitro.

## Genetic manipulation in dictating hepatic-like features

The population of stem cells in the human body possesses a highly different developmental capacity. The process from pluripotent to multipotent and developmentally statuses is attended by total alterations in gene expression. Genes active in previous progenitors are progressively silenced at developmentally later stages, and subsets of cell-type-specific genes are turned off. This development is the result of the careful selective expression of transcription factors together. Hepatocyte differentiation is recognized to be coordinately controlled by the act of multiple hepatocyte nuclear transcription factors, for example, hepatocyte nuclear factor (HNF)-1α and -1β; HNF-3α, -β, and -γ; HNF-4α, HNF-6; and CCAAT/enhancer-binding protein α and β [213]. A liver-enriched transcription factor, HNF-3β, prompts the primary development of the endoderm, the predecessor to produce the liver. HNF-3β in the developing endoderm is essential for the expression of HNF-4α, which shows a potential action in hepatocyte differentiation [213, 214]. Recently, HNF-3β Overexpression has been presented to prompt endoderm differentiation of ES cells. While HNF-3β-transfected ES cells were cultured in hepatocyte-culture medium with FGF-2 in three-dimensional culture, ES cells differentiated into HTC cells with liver-specific metabolic roles [214]. Parp1, (Poly (ADP-ribose polymerase 1)), is an enzyme that catalyzes PARylation, a crucial effector intricate in DNA repair, replication, transcription, and genomic methylation. DNA damage and decreased proliferative responses to mitogens have been detected in hepatocytes from Parp1-deficient mice. Breakdown of Parp1 signaling can exacerbate diet-induced obesity and insulin insensitivity. These findings suggested that Parp1 is an essential element in hepatic protection [215, 216]. Huang et al. demonstrated that Parp1 can improve iPSC generation from somatic cells in the existence of three other reprogramming factors Oct4/Sox2/Klf4 and after stimulation of hepatic differentiation into hepatocyte-like cells. Oct4/Sox2/Klf4-iPSC-Heps expressed liver-specific markers and characteristics, exhibited mature hepatocyte roles [213].

## Conclusion

Alcohol abuse and infection with different viruses, the occurrence of metabolic disorders, carcinoma and injuries are common causes of liver failure. The only curative treatment suggested for liver disease is transplantation that remains expressively restricted by a severe deficiency of organ donors, forcing researchers to use the regenerative medicine modalities to address this issue. Calling attention, producing decellularized scaffolds from the liver organ, 3D bio-printing system and Nano-based 3D scaffolds that simulate the native liver microenvironment seems logical for liver regeneration and to obtain functional hepatic microtissues/organoids. Applying some strategies such as genetic modulation that target small molecules and micro-RNAs could yield an efficient hepatic differentiation rate. Commensurate with these comments, several crucial challenges need to be fully addressed prior to application to the clinical field. For instance, reproducibility in the fabrication of native liver tissue microstructures over microfabrication techniques or self-organization of hepatic organoids remains challenging. Given the complications of cell variations inside the hepatic tissue, appropriate cell density and organization can barely be applied to in vivo milieu. Both juxtacrine and paracrine interactions of non-hepatic cells with hepatocyte regardless 3D bioprinting and decellularized liver scaffolds allow for the reconstruction of ECM at a higher degree. Notably, microtissues/organoids engraftment or whole bioengineered liver transplantation with continuous functionality post-transplantation is challenging. More studies should be made to achieve efficient methods for clinical uses of hepatocytes-derived from stem-cells.

## Data Availability

Data will be available on request.

## Abbreviations

ADSCs: adipose-derived stromal cells
Alb: albumin
BMP: bone morphogenetic protein
BMP4: bone morphogenic protein 4
CNTs: carbon nanotubes
DE: definitive endoderm
DW: direct cell writing
ESCs: embryonic stem cells
EGF: epidermal growth factor
ECM: extracellular matrix
FGF: fibroblast growth factor
FAK: focal adhesion kinase
GelMA: gelatin methacrylate
GAGs: glycosaminoglycans
HSCs: hepatic stellate cells
HGF: hepatocyte growth factor
HNF4α: hepatocyte nuclear factor 4 alpha
HLCs: hepatocyte-like cells
hAECs: human amniotic epithelial cells
hiPSCs: human induced pluripotent cells
hPSCs: human pluripotent stem cells
HA: hyaluronic acid
PEI: hyperbranched polyethyleneimine
iPSCs: induced pluripotent stem cells
ILK: integrin-linked kinase
MSCs: mesenchymal stem cells
MSNs: mesoporous silica nanoparticle
MeHA: methacrylated hyaluronic acid
MAPK: mitogen-activated protein kinase
MKK4: mitogen-activated protein kinase kinase 4
MWNTs: multi-walled-carbon nanotubes
NPCs: non-parenchymal cells
NME2: nucleoside diphosphate kinase B
OSM: oncostatin-M
PCs: parenchymal cells
PLGA: poly lactide-*co*-glycolide
PA: polyacrylamide hydrogel
PEG: polyethylene glycol
PEG-CNTs: polyethylene glycol-linked multi-walled carbon nanotubes
PLA: polylactic acid
RA: retinoic acid
3D: three-dimensional
TGFβ: transforming growth factor β
2D: two-dimensional
VEGF: vascular endothelial growth factor
Wnt: wingless-int
